# Ingestible probes for breath-based monitoring of drug-metabolizing activity from the microbiome

**DOI:** 10.1126/sciadv.aee2632

**Published:** 2026-08-14

**Authors:** Vishal A. Manickam, Carly E. Kimpling, Khoi N. Le, Madeleine E. Troussé, Amy Kang, Aarsh S. Patel, Leslie W. Chan

**Affiliations:** ^1^ Wallace H. Coulter Department of Biomedical Engineering, Georgia Institute of Technology and Emory University, Atlanta, GA 30332, USA.; ^2^ Parker H. Petit Institute for Bioengineering and Biosciences, Georgia Institute of Technology, Atlanta, GA 30332, USA.

## Abstract

Due to their effects on drug activity and safety, drug-metabolizing enzymes (DMEs) from the gut microbiome are attractive drug targets, and noninvasive strategies are needed to measure their activities. Here, we report the development of ingestible probes that sense and produce breath signals for β-glucuronidase (GUS), a microbiome DME that causes drug toxicity. GUS probes comprise safe-to-ingest components and release volatile reporters upon cleavage by GUS. After oral administration, probes transit the gastrointestinal tract intact until they reach the microbiome in the large intestine. There, probes are cleaved by GUS and release volatile reporters, which are rapidly exhaled to provide a near real-time breath signal for GUS activity that can be measured via mass spectrometry. In mouse studies, breath signals were produced in naïve mice with GUS-expressing microbiome but absent in microbiome-depleted mice. Repeated probe dosing and breath analysis enabled monitoring of dynamic changes in GUS activities.

## INTRODUCTION

After administration in the body, most drugs are covalently modified by drug-metabolizing enzymes (DMEs), resulting in metabolites with activity and pharmacokinetics distinct from the parent drug. Drug metabolism has largely been credited to DMEs in the liver (*1*, *2*). However, the gut microbiome produces an arsenal of DMEs, which rivals that of the liver and has garnered attention as potential drug targets (*3*–*12*). One DME that has been of great interest is β-glucuronidase (GUS), which causes off-target drug toxicity in the gastrointestinal (GI) tract (*3*–*10*, *12*). During drug detoxification, a hepatic DME conjugates glucuronic acid (GlcA) to small-molecule drugs to produce inactive, hydrophilic drug metabolites for clearance from the body (*1*). However, when the metabolites are secreted into the GI tract, they encounter GUS, which deconjugates GlcA and causes highly localized drug reactivation in the colon and unwanted toxicity (*3*–*10*, *12*). Notably, GUS causes dose-limiting toxicity for the chemotherapeutic irinotecan, which can ultimately lead to discontinuation of treatment (*8*–*10*, *13*–*19*). In preclinical studies, newly developed GUS inhibitors were able to mitigate irinotecan toxicity (*6*, *8*, *10*). To accelerate the translation of microbiome DME-modulating therapies in humans, tools to monitor the activity of GUS and other DMEs are greatly needed (*6*, *7*, *18*, *20*).

Fluorogenic probes, which fluoresce upon enzymatic cleavage, have been developed to assay GUS and other intestinal enzyme activities ex vivo in stool samples or in vivo via live animal imaging (*21*, *22*). However, real-time measurements are not possible using stool due to its long transit time in the colon, and optical probes are limited to use in small animal models due to light absorption and dispersion in tissues (*23*). Clinically, breath tests have shown great promise, offering a noninvasive method to measure microbial metabolic activity in the GI tract to diagnosis GI diseases (*24*–*31*). Examples include the ^13^C-urea breath test (UBT) for *Helicobacter pylori* infection in the stomach and the small intestinal bacterial overgrowth breath test (SIBO BT) for microbial dysbiosis. The UBT and SIBO BT involve ingestion of ^13^C-urea and lactulose, which are metabolized by the *H. pylori* urease enzyme and microbial fermentation pathways into ^13^CO_2_ and H_2_, respectively, for exhalation (*25*–*28*, *31*–*33*). These examples are part of an emerging paradigm known as induced volatolomics, in which exogenous agents are introduced into the body and converted by disease activity into exhaled volatile organic compounds (VOCs) (i.e., breath biomarkers) (*34*–*40*). This approach obviates the need to rely on naturally occurring breath biomarkers, which have historically been difficult to identify (*41*). Beyond the UBT and SIBO BT, however, the current scope of induced volatolomics is limited by the scarcity of compounds that are known to be metabolized into VOCs by disease-specific activity (*41*).

**Fig. 1.**
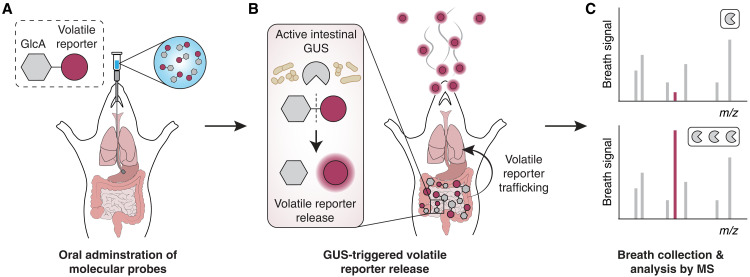
Schematic of the approach. (**A**) GUS probes are synthesized by attachment of a volatile reporter to GlcA via a cleavable O-glycosidic bond. To induce a breath signal for intestinal GUS activity, the probes are delivered via oral gavage. (**B**) After ingestion, probes transit the GI tract intact until they reach the large intestine, where GUS from the gut microbiome cleaves the probes. Probe cleavage triggers the release of the volatile reporters, which recover their characteristic mass and volatility and are exhaled within minutes due to rapid reporter diffusion from the GI lumen to blood and then to the lungs during pulmonary gas exchange. (**C**) Breath signal from exhaled volatile reporters is then measured using mass spectrometry. Together, oral delivery of GUS probes results in breath signals reflecting GUS activity levels in situ.

Here, we report the development of volatile-releasing probes that produce a breath signal for GUS activity after oral delivery (Fig. 1). Ingestible GUS probes were constructed by conjugating a VOC (the volatile reporter) to GlcA (the GUS substrate) through a cleavable glycosidic bond. When conjugated, the reporter is nonvolatile and undetectable. However, when the glycosidic bond is cleaved, the reporter is released and recovers its characteristic mass and volatility for detection as a gas via mass spectrometry. In this work, we administered our probes into mice, which have a GUS-expressing microbiome like humans, to assess breath signal production. We showed that, after oral delivery, probes transit the GI tract intact until reaching the microbiome in the large intestine. Probes are then cleaved by GUS, and liberated reporters follow the same clearance mechanism as naturally produced VOCs, which rapidly diffuse across the intestinal epithelium into systemic circulation and are exhaled after pulmonary gas exchange (*41*). Together, this culminates in a near real-time breath signal for intestinal GUS activity that can be measured via mass spectrometry. Here, we demonstrated that probe-induced breath signals can be used to noninvasively monitor dynamic changes in intestinal GUS activity, which has potential utility for assessment of GUS inhibitor therapies.

## RESULTS

### Development of probes for microbial GUS activity

**Fig. 2.**
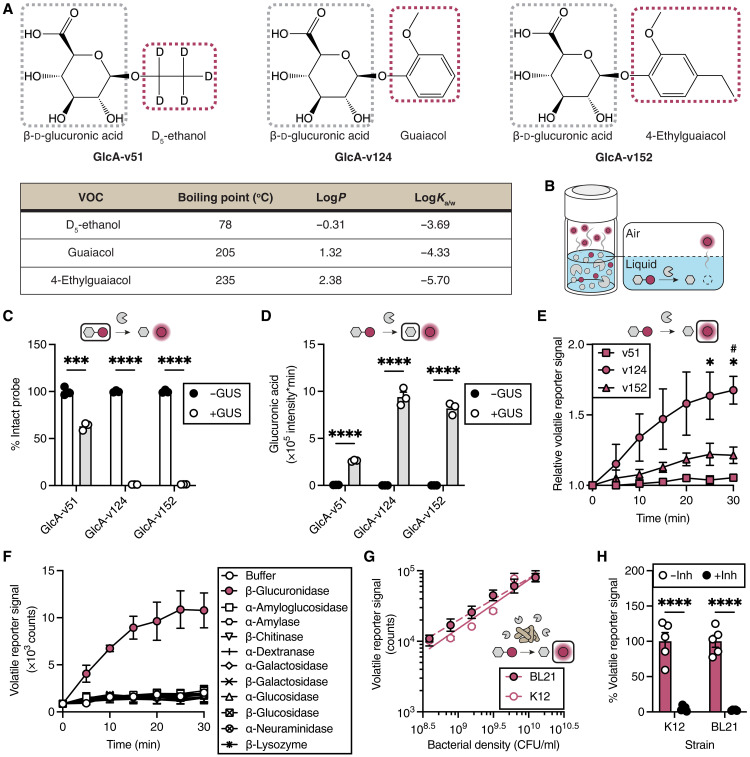
GlcA-v124 produces a volatile signal for microbial GUS activity. (**A**) Chemical structures of GUS probe candidates, which comprise volatile reporters of different structure and physicochemical properties [boiling point (BP), hydrophobicity (Log*P*), and air-water partitioning (Log*K*_a/w_)] conjugated to GlcA. (**B**) Schematic of the cleavage assay used to assess probe activity. In the reaction solution, probe candidates are reacted with recombinant GUS, GUS-containing samples (e.g., microbial cultures), or buffer controls. To determine the extent of probe cleavage, intact probe and GlcA are measured in the reaction solution via liquid chromatography–mass spectrometry (LC-MS). To confirm production of a volatile reporter signal, liberated reporters that have volatilized and partitioned into the reaction headspace are quantified via proton transfer reaction–mass spectrometry (PTR-MS). (**C**) Intact probe and (**D**) GlcA measurements after a 1-hour reaction with recombinant GUS (*n* = 3; unpaired Student’s *t* test, ***P < 0.001 and ****P < 0.0001). (**E**) Volatile reporter signals produced by each probe candidate in response to recombinant GUS activity relative to buffer controls [*n* = 3; two-way analysis of variance (ANOVA) with Dunnett’s multiple comparisons test, *P < 0.05 between v124 and v51 and #*P* < 0.01 between v124 and v152; *N* = 2 independent experiments]. (**F**) Volatile reporter signals produced when GlcA-v124 is reacted with (F) GUS and other recombinant GI glycosidases (*n* = 3; *N* = 2 independent experiments), (**G**) live GUS-producing *E. coli* (*n* = 5; *N* = 2 independent experiments), and (**H**) live *E. coli* in the presence of GUS inhibitor (Inh) (*n* = 5; unpaired Student’s *t* test, ****P < 0.0001; *N* = 2 independent experiments). All data are presented as means ± SD. CFU, colony-forming units.

To engineer GUS probes, VOCs were conjugated to the α-carbon of GlcA via cleavable O-glycosidic linkages. We predicted that the chemical structure of the reporter and its physicochemical properties would affect probe cleavage kinetics, reporter biodistribution, and, ultimately, the magnitude of reporter signal in breath. Therefore, three GUS probes with different reporters were synthesized to investigate the effect of reporter properties on breath signal (Fig. 2A). D_5_-ethanol, guaiacol, and 4-ethylguaiacol were selected to represent aliphatic and aromatic reporter structures and a range of boiling points (BPs), hydrophobicities (Log*P*), and air-water partition coefficients (Log*K*_a/w_). VOCs with low BPs and high air-water partition coefficients readily vaporize into gas phase. All reporters were selected for their generally recognized as safe (GRAS) US Food and Drug Administration designation. GRAS compounds are safe to ingest and are found naturally in food or commonly used as food additives to provide scent and flavor. GlcA itself is an oxidized form of glucose that is produced from glucose metabolism and, as previously discussed, is critical for detoxification and removal of drugs and other xenobiotics from the body (*42*, *43*). Therefore, GUS probes comprise safe and ingestible components, minimizing the barrier to clinical translation. In this work, probes will be referred to as GlcA-v*x*, where *x* indicates the mass of the volatile reporter. Probes with D_5_-ethanol, guaiacol, and 4-ethylguaiacol reporters (i.e., GlcA-v51, GlcA-v124, and GlcA-v152, respectively) were successfully synthesized as confirmed via proton nuclear magnetic resonance (1H-NMR) and liquid chromatography–mass spectrometry (LC-MS; figs. S1 to S4).

### GlcA-v124 produces a volatile signal for GUS activity

After probe synthesis, we sought to determine whether the probes could sense and produce a volatile reporter signal for GUS activity. GUS from *Escherichia coli* has superior catalytic efficiency compared to GUS from other bacterial species and is the primary driver of irinotecan toxicity (*4*, *44*). Therefore, in cleavage assays, we reacted each probe with recombinant GUS from *E. coli* and measured solubilized and gaseous reaction products using LC-MS and proton transfer reaction–mass spectrometry (PTR-MS), respectively (Fig. 2B). Reaction with GUS for 1 hour resulted in some or complete disappearance of the intact probe and corresponding appearance of GlcA in solution (Fig. 2, C and D). These results confirm that GUS is able to cleave the O-glycosidic bond tethering the reporters to GlcA. Less GlcA-v51 was cleaved than the other probes (37% for GlcA-v51 versus >99% for GlcA-v124 and GlcA-v152) (Fig. 2C). This suggests that aromatic reporters (i.e., guaiacol and 4-ethylguaiacol) allow probes to bind the catalytic pocket of GUS more favorably for probe cleavage than aliphatic reporters (i.e., D_5_-ethanol).

Volatile reporter signal is ultimately a product of both probe cleavage and volatilization of the liberated reporter into the reaction headspace. While a probe may be cleaved efficiently (as confirmed by LC-MS), it may not produce strong reporter signal if the liberated reporter remains in solution rather than vaporizing into the reaction headspace and vice versa. Comparison of GlcA-v124 and GlcA-v152 illustrates this perfectly. Both probes were completely cleaved after a 1-hour reaction (Fig. 2C), but GlcA-v124 produced a significantly greater volatile reporter signal than the other probes by 25 and 30 min after the start of the reaction (Fig. 2E). We attribute this to guaiacol’s superior volatility (i.e., 23-fold greater *K*_a/w_) over that of 4-ethylguaiacol. GlcA-v51 is an example of the reverse. While D_5_-ethanol has the most favorable volatility of the three reporters (i.e., lowest BP and highest Log*K*_a/w_), GlcA-v51 was cleaved less efficiently than the other probes (Fig. 2C) and ultimately produced the lowest volatile reporter signal when reacted with GUS (Fig. 2E). Additionally, ionization efficiency of the reporters in the mass spectrometer may also contribute to differences in resulting signal. Together, these results underscore the importance of reporter structure and volatility for producing a functional probe. The combined effect of probe cleavage and reporter volatilization is reflected in the apparent catalytic efficiencies ($k_{\text{cat}}^{\text{app}}/K_{m}^{\text{app}}$) for each probe, which were derived from volatile reporter signals (Table 1 and fig. S5). Because signal is dependent on more than simple substrate cleavage kinetics, we refer to the derived values as the apparent turnover number ($k_{\text{cat}}^{\text{app}}$) and apparent Michaelis constant ($K_{m}^{\text{app}}$). Overall, GlcA-v124 exhibited the greatest apparent catalytic efficiency, producing signal for GUS activity 11 and 35 times more efficiently than GlcA-v51 and GlcA-v152, respectively. Therefore, subsequent studies focused on further characterization of GlcA-v124.

**Table 1.** Catalytic efficiency of GUS for GlcA-v51, GlcA-v124, and GlcA-v152. The values represent the means (±SD) of three individual experiments.

Probe	$k_{\text{cat}}^{\text{app}}$ [counts s^−1^ mM^−1^ (×10^4^)]*	$K_{m}^{\text{app}}$ (mM)	$k_{\text{cat}}^{\text{app}}/K_{m}^{\text{app}}$ [counts s^−1^ (×10^4^)]*
GlcA-v51	5.06 (±0.28)	0.92 (±0.06)	5.51 (±0.14)
GlcA-v124	159.38 (±13.97)	2.58 (±0.14)	61.73 (±3.04)
GlcA-v152	2.57 (±0.77)	1.51 (±0.55)	1.74 (±0.30)

* Counts is a unit of volatile reporter abundance.

For optimal signal-to-noise ratio (SNR) in breath, probes should be resistant to cleavage by off-target glycosidases in the GI tract. To assess specificity of GlcA-v124 for GUS activity, in vitro cleavage assays were completed (Fig. 2F). Incubation with GUS led to a rapid increase in reporter signal. In contrast, GlcA-v124 did not produce any signal when incubated with other glycosidases, including α-amylase, which is highly abundant in the upper GI tract and has broad substrate specificity (*45*). Volatile reporter signals produced by GlcA-v124 were also directly proportional to GUS concentration, confirming the probe’s ability to sense a range of GUS activities (fig. S6). To better simulate in vivo conditions, we incubated GlcA-v124 with live GUS-producing bacteria, *E. coli* strains K12 and BL21. In both strains, we observed a strong linear correlation between bacterial density and volatile reporter signal [Fig. 2G, coefficient of determination (*r*^2^) > 0.9]. Introduction of a GUS inhibitor, UNC10201652 (*6*), into K12 and BL21 cultures with GlcA-v124 attenuated all reporter signal, further confirming probe specificity for GUS activity (Fig. 2H). Together, these results confirm that conjugation of guaiacol to GlcA yields a highly sensitive and specific probe for microbial GUS activity.

### GUS probes are stable in transit before reaching the cecum

**Fig. 3.**
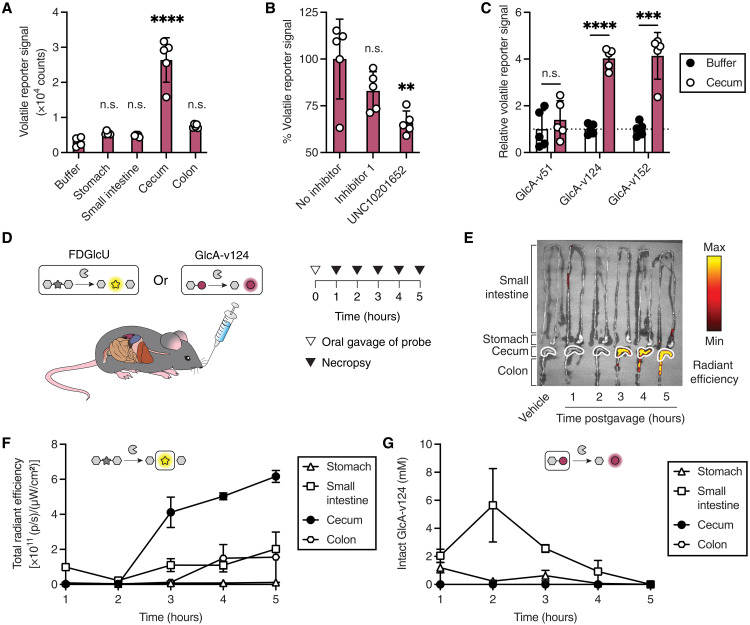
GUS probes are stable in transit and first enter the cecum 2 to 3 hours after oral gavage. (**A**) Volatile reporter signal produced by GlcA-v124 when it is reacted with homogenates of the mouse stomach, small intestine, and large intestine [separated into the cecum and colon) (means ± SD, *n* = 5; one-way ANOVA with Dunnett’s multiple comparisons test, **P < 0.01, ****P < 0.0001, and not significant (n.s.); *N* = 2 independent experiments]. (**B**) Attenuation of GlcA-v124 reporter signal in cecum homogenates using GUS inhibitors (inhibitor 1 or UNC10201652) (means ± SD, *n* = 5; one-way ANOVA with Dunnett’s multiple comparisons test, **P < 0.01, ****P < 0.0001, ns = not significant; *N* = 2 independent experiments). (**C**) Comparison of volatile reporter signals produced by GlcA-v51, GlcA-v124, and GlcA-v152 when reacted with cecum homogenates relative to buffer controls (means ± SD; unpaired Student’s *t* test *n* = 5, ns = not significant, ***P < 0.001, ****P < 0.0001). (**D**) Schematic of the probe pharmacokinetic studies used to identify the window of time during which probes are in the large intestine and cleaved by GUS. Naïve mice were administered fluorescein-di-β-d-glucuronide (FDGlcA) or GlcA-v124 via oral gavage and euthanized at hourly time points. Major GI organs were excised and imaged for fluorescence signal, which indicates FDGlcA cleavage by GUS, or were homogenized to quantify intact GlcA-v124 via LC-MS. (**E**) Representative IVIS images of the major GI compartments and (**F**) corresponding quantification of fluorescent signal for each GI compartment over time (means ± SEM, *n* = 3; *N* = 2 independent experiments). (**G**) Concentrations of GlcA-v124 in the major GI compartments over time (means ± SEM, *n* = 3).

Like humans, mice have GUS activity in the large intestine from the microbiome (*6*, *8*, *22*, *46*, *47*). Mechanisms for VOC elimination in breath are also conserved in humans and rodents (*48*, *49*). Therefore, naïve mice were used to determine whether oral delivery of GUS probes would induce breath signals for intestinal GUS activity. Before completing breath studies, we first assessed GlcA-v124 stability in homogenates of the mouse stomach, small intestine, cecum (a pouch at the start of the large intestine), and colon (the remaining part of the large intestine). In the mouse, the cecum houses the highest density of microbes (∼3 times greater than that of the colon) and is, therefore, a particularly metabolically active portion of the large intestine (*50*). Reporter signal was only produced in cecum homogenates (Fig. 3A). The absence of signal from the colon may be explained by the lower microbial density compared with the cecum. Furthermore, rinsing of the colon to prevent assay interference from fecal pellets may have removed microbes loosely associated to the mucosa. Cecum reporter signal was partially attenuated with addition of UNC10201652 and another GUS inhibitor (i.e., inhibitor 1) (Fig. 3B), confirming that, at least, part of the signal is due to probe cleavage by GUS. UNC10201652 and inhibitor 1 were designed to specifically inhibit loop 1 GUS architectures produced by *E. coli* (*6*, *20*), which we observed firsthand in *E. coli* cultures studies (Fig. 2H). Therefore, we attributed the remaining signal to uninhibited GUS activity from other bacterial species. Together, these results indicate that GlcA-v124 is resistant to premature degradation before reaching the cecum, where it is cleaved by GUS. In a comparison of the three probes in cecum homogenates, we again observed negligible signal from GlcA-v51 and strong signals from Glc-v124 and Glc-v152 (Fig. 3C). LC-MS analysis further confirmed more extensive cleavage of GlcA-v124 and GlcA-v152 than GlcA-v51 in cecum homogenates (76 and 85% probes compared to 49%, respectively) (fig. S7).

Due to the rapid volatile trafficking from the GI lumen to breath [on the order of minutes; (*41*)], reporter signal in breath provides a near real-time readout for GUS activity. Therefore, breath should be sampled when probes are in the cecum, where the GUS activity is localized. To identify this window of time, we used a commercially available fluorogenic GUS probe, fluorescein-di-β-d-glucuronide (FDGlcU), which produces a strong fluorescence signal when GlcA is removed (*22*). For this study, mice were euthanized at hourly intervals of 1 to 5 hours after oral gavage of probes for imaging of the GI tract via the In Vivo Imaging System (IVIS) (Fig. 3D). Fluorescence signal was first observed in the cecum 3 hours postadministration, indicating that FDGlcU first entered the cecum 2 to 3 hours after oral gavage (Fig. 3, E and F). Fluorescence signal in the cecum continued to increase from 3 to 5 hours, with signal also appearing in the colon during this time. These results support the transit of probes from the small intestine to the cecum and proximal colon 2 to 5 hours after oral gavage. This and subsequent studies were completed with fasted mice to minimize interference from food boluses in the GI tract and reduce background signal from dietary volatiles (*51*, *52*).

Different small molecules can vary in their GI transit times as a function of their hydrophobicity (*53*, *54*). Therefore, we also used LC-MS to quantify intact GlcA-v124 in tissue homogenates of the mouse stomach, small intestine, cecum, and colon 1 to 5 hours after oral delivery (Fig. 3D). Due to high levels of endogenous GlcA in the GI tract, GlcA measurements could not be used to determine the abundance of cleaved GlcA-v124. GlcA-v124 concentrations reflected the transit of GlcA-v124 through the upper GI tract (Fig. 3G). One to 2 hours after oral gavage, declining probe concentrations in the stomach corresponded to rising concentrations in the small intestine. Based on the steady decline of GlcA-v124 concentrations in the small intestine between 2 and 5 hours, we expected GlcA-v124 to appear in the GI compartments downstream of the small intestine within that window of time. However, no probe was detected in the cecum and colon, which we attributed to rapid and exhaustive cleavage of the probe in those compartments. Together, these results suggest that the optimal breath collection time should fall between 2 and 5 hours after probe administration in mice.

### GUS probes induce a breath signal after oral delivery

**Fig. 4.**
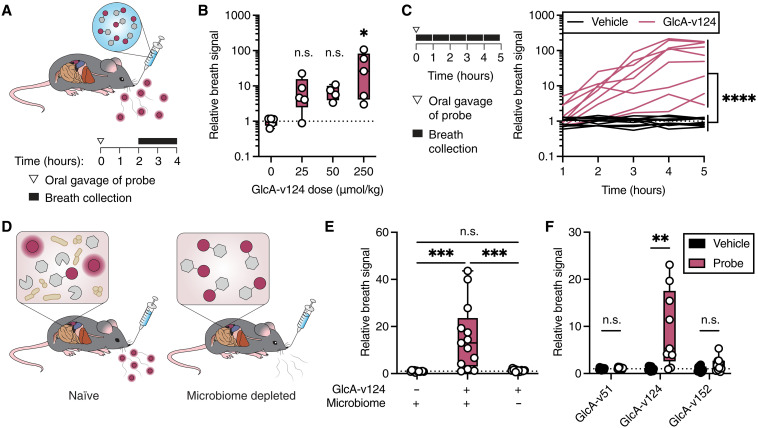
GlcA-v124 induces breath signals for microbial GUS activity after oral delivery. (**A**) Schematic of the breath studies used to investigate breath signal production after GlcA-v124 administration. Unless specified, breath was collected over a 2-hour duration 2 to 4 hours after probe delivery. (**B**) Breath signals induced by different GlcA-v124 doses [*n* = 4 to 5; one-way ANOVA with Dunnett’s multiple comparisons test, *P < 0.05 and not significant (n.s.)]. (**C**) Breath signal kinetics after oral delivery of GlcA-v124 (250 μmol/kg; *n* = 9 to 10; two-way ANOVA, ****P < 0.0001; *N* = 2 independent experiments). (**D**) Schematic showing expected differences in breath signal in naïve mice versus microbiome-depleted mice. (**E**) Breath signals in naïve mice with intact microbiome versus in microbiome-depleted mice [*n* = 12 to 14; one-way ANOVA with Tukey’s multiple comparisons test, ***P < 0.001 and not significant (n.s.)]. (**F**) Comparison of breath signals induced by GlcA-v51, GlcA-124, and GlcA-v152 [*n* = 9 to 10; unpaired Student’s *t* test, **P < 0.01 and not significant (n.s.); *N* = 2 independent experiments]. All box-and-whisker plots show median and range.

Next, we assessed the ability of probes to induce breath signals for intestinal GUS activity. For these studies, GlcA-v124 or a vehicle control was administered via oral gavage to naïve C57Bl/6 mice, which have GUS-expressing microbiome (Fig. 4A) (*6*, *8*, *20*, *55*). Mice were then placed in a sealed chamber for breath collection. Airflow was continuously directed through the chamber to displace the headspace into sorbent tubes for volatile reporter collection. Sorbent tubes contain adsorbent materials for VOCs and were used to concentrate reporters from large volumes of mouse breath for more sensitive detection. Unless otherwise specified, Tenax TA sorbent tubes, which capture a diverse range of VOCs and are commonly used in human breath analysis, were used with an air flow rate of 100 ml/min, the fastest flow rate that did not impair guaiacol adsorption in the sorbent tubes (fig. S8).

In initial studies, we sought to identify a minimum probe dose that would produce breath signals. Based on the pharmacokinetic studies, we expected that breath signal, if produced, would initially appear 2 to 3 hours after probe delivery. Due to smaller expiratory volumes of mice compared with humans, breath was collected 2 to 4 hours after oral gavage. In humans, we expect to observe differences in breath signals by integration across a smaller window of time. While no breath signal was observed at probe doses of 25 and 50 μmol/kg, a pronounced breath signal was observed in mice administered the probe at 250 μmol/kg (Fig. 4B). Using this dose, we extended our time points to investigate breath signal kinetics. Breath was sampled for hour-long durations from 0 to 5 hours after probe administration and revealed signal kinetics that corresponded to the probe pharmacokinetics (Fig. 4C). Breath signals showed the sharpest increase 2 to 4 hours after GlcA-v124 ingestion, the window of time during which the probe enters and is cleaved in the cecum. This was followed by a plateau in signal. To confirm that breath signal was due to probe metabolism by microbial GUS, we compared breath signals produced in naïve mice and microbiome-depleted mice. We expected that microbiome clearance would eliminate intestinal GUS activity and, therefore, prevent probe cleavage (Fig. 4D). The microbiome was depleted via prolonged antibiotic treatment, and loss of intestinal GUS activity was confirmed ex vivo (fig. S9). As expected, breath signal was attenuated in microbiome-depleted mice (Fig. 4E), confirming the role of the microbiome in breath signal production.

After verifying successful breath signal production, we investigated whether the superior catalytic efficiency of GlcA-v124 (Table 1) translated to superior breath signal compared with that of GlcA-v51 and GlcA-v152. Only GlcA-v124 produced a breath signal (Fig. 4F). Despite strong reporter signal produced in reactions with recombinant GUS and cecum homogenates (Figs. 2E and 3C), GlcA-v152 failed to produce breath signal after oral delivery. We attributed this to the high lipophilicity and low air-water partitioning of 4-ethylguaiacol, which lead to reporters sequestered in tissues instead of cleared in breath. Breath signal was also absent for GlcA-v51 when breath volatiles were captured using Carbotrap 217, a multibed sorbent tube that is more suitable for collecting light, polar VOCs like D_5_-ethanol (fig. S10). Together, these results confirm that oral delivery of volatile-releasing probes can be used to induce breath signals for intestinal GUS activity. Furthermore, these results underscore the importance of volatile reporter choice for generating breath signals.

### Breath signals enable real-time monitoring of GUS activity

**Fig. 5.**
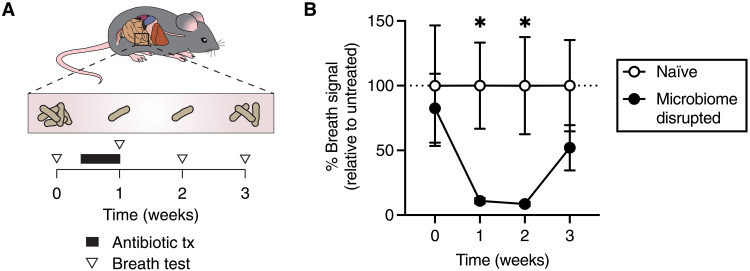
Probe-induced breath signals can be used to monitor dynamic changes in microbial GUS activity. (**A**) Schematic of the expected bacterial abundance before microbiome depletion (*t* = week 0), immediately after microbiome depletion (*t* = week 1), and during microbial recolonization in the absence of antibiotic treatment (tx; *t* = weeks 2 and 3). Each breath test comprises GlcA-v124 administration followed by PTR-MS analysis of breath samples collected 2 to 4 hours after probe ingestion. (**B**) Loss and recovery of breath signal over time due to depletion and recovery of the gut microbiome [means ± SEM, *n* = 7 to 9; unpaired Student’s *t* test, *P < 0.05 and not significant (n.s.)].

A benefit of breath testing is the ability to monitor dynamic changes in metabolic activity, which has potential utility for monitoring DME-modulating treatments. In microbiome-depleted mice, the cessation of antibiotic treatment causes bacterial recolonization of the gut and restoration of intestinal GUS activity (Fig. 5A). Therefore, antibiotic-treated mice were used to model the dynamic range of intestinal GUS activities and to assess the utility of GlcA-v124 for monitoring GUS activity levels. In these studies, each breath test is defined as an oral dose of Glc-v124 followed by analysis of a breath sample collected 2 to 4 hours after probe delivery. When four total breath tests were completed at weekly intervals before, during, and after antibiotic treatment, the resulting breath signals captured the loss and recovery of GUS activity corresponding with microbiome depletion and recolonization (Fig. 5B). In summary, these results highlight the utility of GlcA-v124 for monitoring changes in intestinal GUS activity via simple breath testing.

## DISCUSSION

Due to their impact on drug activity and safety, microbiome DMEs are a compelling potential class of druggable targets, and noninvasive tools are greatly needed to measure their activities (*3*–*10*, *12*). GUS, in particular, is a DME of interest because it causes drug-induced GI toxicity via drug reactivation in the large intestine (*3*–*10*, *12*). We developed ingestible probes to enable measurement of intestinal GUS activity via a simple breath test. GUS probes were developed by linking edible components, GlcA (a glucose derivative) and GRAS VOCs (which are used as food flavorings), through a cleavable O-glycosidic bond. In mice, we showed that probe ingestion induces breath signals for GUS activity from the gut microbiome. We also demonstrated that probes can be dosed at multiple time points to capture dynamic changes in GUS activity via breath analysis, which has potential utility for assessment of new GUS inhibitor therapies.

By engineering breath signals de novo, we eliminate the need for breath biomarker discovery. We and others have demonstrated the versatility of the induced volatolomics approach for detection of lung diseases and cancer (*35*, *37*, *38*, *40*). Using this approach, we can optimize the SNR of breath signals by fine-tuning parameters such as substrate/reporter selection, administration route, and probe dose. As demonstrated here, reporter selection proved critical for breath signal production due to its effect on probe cleavage kinetics and reporter clearance from the body. We also used GRAS VOCs as reporters, which are safe to ingest and ideal for clinical translation. Using allometric scaling, we find that the GlcA-v124 dose used in mouse studies yields a human equivalent dose comparable to dosing that is used clinically for the UBT (*56*–*58*). Assuming complete liberation of guaiacol from the ingested probes, the resulting concentrations would still be safely within the limit for repeated dose toxicity (*59*). While GRAS VOCs are found in foods, background signal from diet can be minimized via overnight fasting, which is already implemented for the UBT and SIBO breath tests. Furthermore, interindividual variability in background signal due to prolonged VOC exposure via other sources (e.g., cosmetic and cleaning products and the environment) can be accounted for by collecting breath samples before and after probe ingestion to determine the absolute change in breath signal.

Beyond DMEs, the gut microbiome produces hundreds of distinct enzymes that are not encoded in the human genome that critically affect nutrient metabolism, host immunity, and neurological and cardiovascular health (*11*, *60*–*67*). While metagenomic sequencing is the most-frequented tool to study the microbiome, it only measures enzyme gene abundance, which does not necessarily correlate to enzyme activity levels due to regulation at the posttranscriptional level. Given the modular structure of our probes, we expect that the substrate component can be readily exchanged to produce breath signals for diverse microbial and host enzyme activities in the GI tract. Ultimately, engineered breath signals can serve as functional biomarkers, enabling noninvasive detection of GI diseases.

## MATERIALS AND METHODS

### Animals

Six- to 9-week-old female C57BL/6J mice were purchased from the Jackson Laboratory (strain no. 000664). All animal procedures were approved by the Georgia Institute of Technology Institutional Animal Care and Use Committee (CHAN-A100472).

### GUS probe synthesis

GlcA-v124 and GlcA-v152 were synthesized by WuXi AppTec. Briefly, guaiacol or 4-ethylguaiacol was reacted with O-acetylated GlcA with a bromine or trichloroacetimidate leaving group in the presence of Ag_2_O or BF_3_/Et_2_O, respectively. Intermediate products were purified using chiral chromatography to enrich for the β-stereoisomer before deprotection with sodium hydroxide. Intermediate and final products were analyzed for purity by 1H-NMR, two-dimensional nuclear magnetic resonance (2D-NMR), high-performance liquid chromatography, and tandem mass spectrometry (MS/MS), and the stereoisomer confirmed by measuring the *^1^J*_*C1*,*H1*_ coupling frequencies (*68*). GlcA-v51 was purchased from Smolecule (catalog no. S875950).

### In vitro cleavage assays

Probes were reacted with recombinant glycosidases in Exetainer vials (Labco Limited) at a final concentration of 100 μM probe and 100 nM glycosidase in 1× phosphate-buffered saline (PBS; pH 7.0 to 7.4). Vials contain a rubber septum cap, and headspace sampling was completed by piercing the septum with the needle of a gastight syringe (Hamilton) and pulling the plunger back to draw the desired volume. To quantify volatilized reporters produced from the reaction, 100 μl of reaction headspace was sampled immediately after starting the reaction and every 5 min thereafter for up to 1 hour and immediately injected into a PTR-TOF 1000 Ultra (Ionicon). Volatile signals were processed using PTR-MS Viewer V3.4.2.7 (Ionicon), and peak areas were computed using a custom Python script before plotting values using GraphPad Prism 10 software. Intact probes and solubilized reaction products were quantified using LC-MS; more specifically, a Vanquish Horizon UHPLC system (Thermo Fisher Scientific) equipped with an ACQUITY UPLC BEH Amide column (Waters Corp, 2.1 mm by 100 mm, 1.7 μm) coupled to an Orbitrap Exploris 240 mass spectrometer (Thermo Fisher Scientific). Before analysis, the reaction solution was diluted 1:4 in methanol and centrifuged at 10,000*g* for 10 min to precipitate and remove glycosidases and other proteins from the sample. The supernatant was stored at −20°C until analysis. The LC-MS mobile phases were 80:20 water:acetonitrile with 10 mM ammonium formate and 0.1% formic acid (mobile phase A) and acetonitrile with 0.1% formic acid (mobile phase B). The chromatographic method used the following gradient program: 0 min, 5% A; 0.5 min, 5% A; 8 min, 60% A (curve 7); 9.4 min, 60% A; 9.5 min, 5% A; and 12 min, 95% A. The flow rate was set at 0.40 ml/min. The column temperature was set to 40°C, and the injection volume was 1 μl. Full-scan data were acquired in positive mode with a filter of 100 to 1000 mass/charge ratio (*m*/*z*) at 60,000 resolution. Targeted MS/MS was collected on the probes and cleavage products with an isolation window of 1.0 *m*/*z*, normalized higher-energy collisional dissociation (HCD) collision energy of 70%, and an Orbitrap resolution of 30,000. Xcalibur v4.5.474.0 (Thermo Fisher Scientific) was used to process the raw LC-MS data. Measured signal intensities for each species were integrated with respect to time to quantify peak area.

### Bacterial culture assays

K12 (New England Biolabs, catalog no. E4104) and BL21 (Thermo Fisher Scientific, catalog no. EC0114) *E. coli* strains were cultured overnight in Luria-Bertoni broth. Secondary cultures were prepared using 1:50 dilutions of overnight cultures in fresh medium and cultured for 3 hours to an optical density at 600 nm (OD_600_) of 1 for log-phase growth, as measured by NanoDrop One-c (Thermo Fisher Scientific). Cultures were centrifuged at 3000*g* for 10 min to pellet bacteria. Bacteria were washed twice with sterile 1× PBS (pH 7.0 to 7.4) and resuspended to specific bacterial densities using a conversion factor of 8 × 10^8^ colony-forming units/ml per 1 OD_600_. For GUS inhibition assays, UNC10201652 (Sigma-Aldrich, catalog no. SML3236) was initially dissolved in dimethyl sulfoxide (DMSO) to 4.9 mM before further dilution in deionized water with 1% (v/v) Tween 80 (TCI America, catalog no. T0546). Bacterial cultures were treated with diluted UNC10201652 inhibitor or an equal volume of DMSO in 1% Tween 80 solution for 1 hour before adding probes for a final concentration of 100 μM inhibitor and 100 μM probe. Reactions proceeded at room temperature for 1 hour on an orbital shaker before 1-ml reaction headspace was sampled using a gastight syringe for PTR-MS quantification. Areas under the curve (AUCs) were determined for each injection and plotted using GraphPad Prism 10 software.

### Ex vivo cleavage assays

GI tract organs were collected from healthy C57BL/6J mice. Small intestines were flushed with ice cold PBS to remove contents, and contents of the colon were manually removed to minimize interference of the food bolus and fecal pellets in assessing tissue and mucosal cleavage activity. Organs were placed into 2-ml Safe-Lok Eppendorf tubes with 3.2- to 5-mm stainless steel beads and homogenized in 500 μl of 100 mM phosphate (pH 7.0) with 1× protease inhibitor cocktail at 30 Hz for 10 min using a TissueLyzer (QIAGEN). Homogenates were centrifuged at 10,000*g* for 5 min, and supernatant was collected. Centrifugation was repeated once more to remove tissue debris to produce clarified homogenates, which were stored at −80°C. Protein concentrations were determined using bicinchoninic acid (BCA) quantification (Thermo Fisher Scientific). Probes were reacted with homogenates in Exetainer vials at a final homogenate concentration of 500 to 1000 μg/ml in 1× PBS (pH 7.0 to 7.4) and 100 μM probe. Reaction headspace (1 ml) was sampled using gastight syringes after 1 hour of incubation on an orbital shaker for PTR-MS quantification. Soluble products were quantified by LC-MS. For GUS inhibition assays, UNC10201652 or inhibitor 1 (Sigma-Aldrich, catalog no. 347423) were initially dissolved in DMSO then diluted in 1% Tween 80 (v/v) in deionized water for a final concentration of 100 μM. Tissue homogenates were treated with diluted inhibitor or an equal volume of DMSO diluted in 1% Tween 80 for at least 1 hour. For GUS activity measurements using a commercially available fluorogenic probe (4-methylumbelliferyl-β-d-glucuronide; Cayman Chemical, catalog no. 17203), 10 μM probe was reacted with clarified homogenates diluted to protein (100 μg/ml) in 100 mM phosphate (pH 7.0) at 37°C in a 384-well plate. Fluorescence signals were measured using a spectrophotometer (BioTek Synergy H4 Microplate Reader) every 5 min for 30 min at excitation and emission wavelengths of 372 and 445 nm, respectively. Background signal from substrate only controls were subtracted from cleavage reactions at each time point before performing a linear regression to calculate the slope cleavage rate using GraphPad Prism 10.

### FDGlcU pharmacokinetics study

A fluorescein-di-β-d-glucuronide (FDGlcU; Thermo Fisher Scientific) solution (1 mg/ml) was prepared in deionized (DI) H_2_O. FDGlcU solution (100 μl) was delivered via oral gavage into healthy mice, and mice were euthanized at 1, 2, 3, 4, and 5 hours after probe administration. Fluorescence signal in excised GI organs was imaged using IVIS Spectrum imager, and signals were quantified by signal integration in each organ using the IVIS Spectrum imager software.

### GlcA-v124 pharmacokinetics study

One hundred microliters of 50 mM GlcA-v124 in DI H_2_O was delivered via oral gavage into healthy mice, and mice were euthanized at 1, 2, 3, 4, and 5 hours after probe administration. Excised GI compartments were scraped to collect luminal contents. Each sample (50 mg) was homogenized with 3.2- to 5-mm stainless steel beads in 1.6 ml of 80% methanol in DI H_2_O using the TissueLyzer (30 Hz, 10 min). Homogenates were centrifuged at 21,100*g* for 5 min before 1 ml of supernatant was transferred to a new set of tubes. Methanol and DI H_2_O were completely evaporated from samples using a CentriVap (Labconco) before reconstitution in 50 μl of 80% methanol in DI H_2_O to concentrate samples. Intact GlcA-v124 abundance was determined by LC-MS as previously described, and concentrations were calculated using a standard curve of pure GlcA-v124 in 80% methanol in DI H_2_O.

### Breath studies

Healthy mice were administered 0.5 to 5 μmol of GlcA-51, GlcA-v124, or GlcA-v152 in 100 μl of DI H_2_O via oral gavage and immediately placed in breath collection chambers (Braintree Scientific, catalog no. EZ-17 7), which are connect to a medical-grade air tank (Airgas, catalog no. AI USP300) at the chamber inlet and to sorbent tubes [Carbotrap 217 (Supelco, catalog no. 29744-U) and Tenax TA (Supelco, catalog no. 20010-U)] at the chamber outlet. Air (50 to 150 ml/min) was flowed continuously through chambers to collect breath onto sorbent tubes for 1 to 2 hours. Sorbent tubes were stored at 4°C until analysis. Before analysis, tubes were dry purged with pure nitrogen gas at 100 ml/min for 10 min at ambient temperature to remove moisture. To thermally desorb volatile reporters from the sorbent tubes and direct them into the mass spectrometer for quantification, sorbent tubes were heated in an aluminum heating block to 80°C, and nitrogen was flowed through sorbent tubes for 5 min at 35 (±5) ml/min into the inlet of the PTR-MS. Signals peaks for protonated D_5_-ethanol (m52), protonated guaiacol (m125), and protonated 4-ethylguaiacol (m153) *m*/*z* channels were integrated to determine total counts. Integrated VOC reporter signals were then normalized to hydronium abundance before quantifying fold change over vehicle gavage baseline following the equation below.

$$\text{Relative breath signal}=\frac{\left({\text{Counts}}_{\text{VOC},\text{Probe}}/{\text{Counts}}_{H3O+,\text{Probe}}\right)}{\left({\text{Counts}}_{\text{VOC},\text{Vehicle}}/{\text{Counts}}_{H3O+,\mathrm{Vehicle}}\right)}$$

### Microbiome depletion for breath studies

Mice were treated with a prolonged course of orally administered antibiotics as previously described (*22*). For 4 days before breath testing, ampicillin (1 g/liter; VWR, catalog no. 97061-442) was administered ad libitum in drinking water, and 100 μl containing neomycin (20 mg/ml; Chem-Impex, catalog no. 00224) and vancomycin (10 mg/ml; VWR, catalog no. 97062-554) was administered via oral gavage two times a day. For longitudinal monitoring studies, breath tests were completed at weekly intervals. Each breath test involved oral administration of 5 μmol of probe and breath collection 2 to 4 hours after probe administration.

### Statistical analysis

All statistical analysis was completed in GraphPad Prism 10.4.1 software. Statistical tests and sample sizes are described in the figure and table captions. Shapiro-Wilk test was performed to assess normality of datasets to determine the appropriate statistical test. Robust regression and outlier removal (ROUT) method (*Q* = 1%) was used to identify outliers for in vivo studies.
