# Supplementary material for: Ingestible probes for breath-based monitoring of drug-metabolizing activity from the microbiome
Source: Sci Adv. 2026 Aug 14;12(33):eaee2632. doi: 10.1126/sciadv.aee2632 (PMC13475483; doi:10.1126/sciadv.aee2632)
Supplement: Supplementary file 1 [file sciadv.aee2632_sm.pdf]

Science Advances
AAAS

Supplementary Materials for

Ingestible probes for breath-based monitoring of drug-metabolizing activity from the microbiome

Vishal A. Manickam *et al.*

Corresponding author: Leslie W. Chan, leslie.chan@gatech.edu

Sci. Adv. **12**, eaee2632 (2026)
DOI: 10.1126/sciadv.aee2632

The PDF file includes:

Figs. S1 to S10
Legend for data S1

Other Supplementary Material for this manuscript includes the following:

Data S1

Supplementary Figures

A

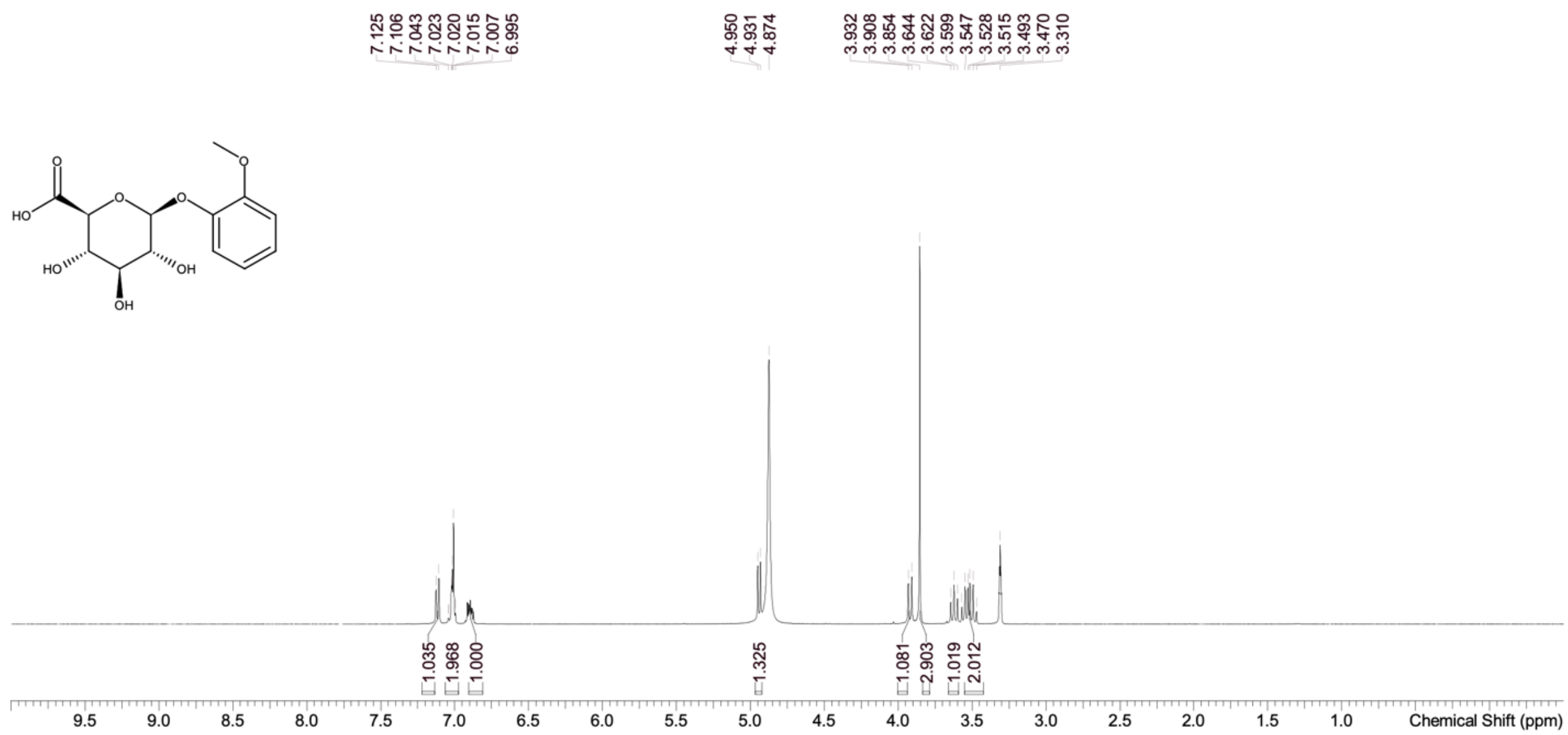

B

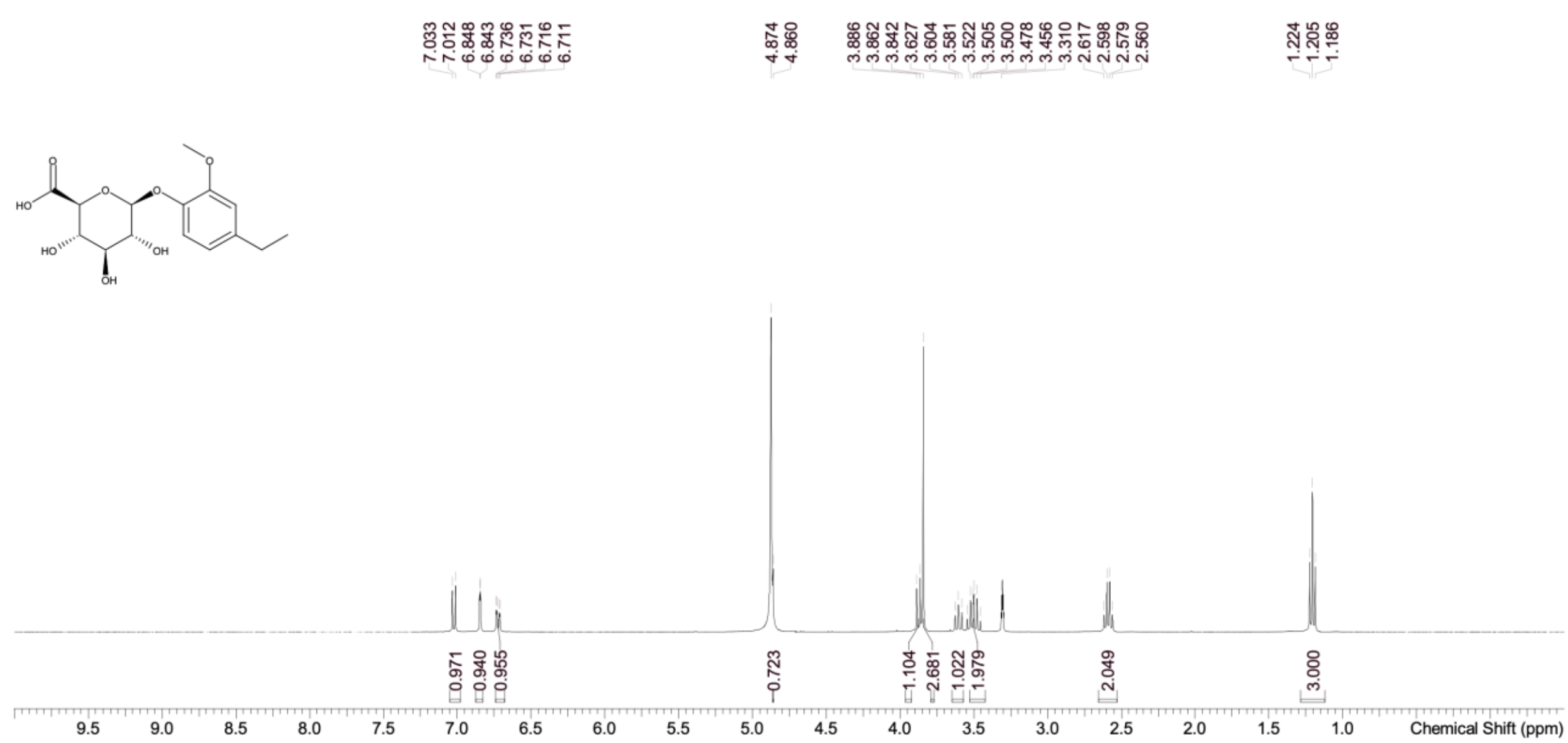

Fig. S1. HNMR spectra to confirm the chemical structure of (A) GlcA-v124 and (B) GlcA-v152.

A

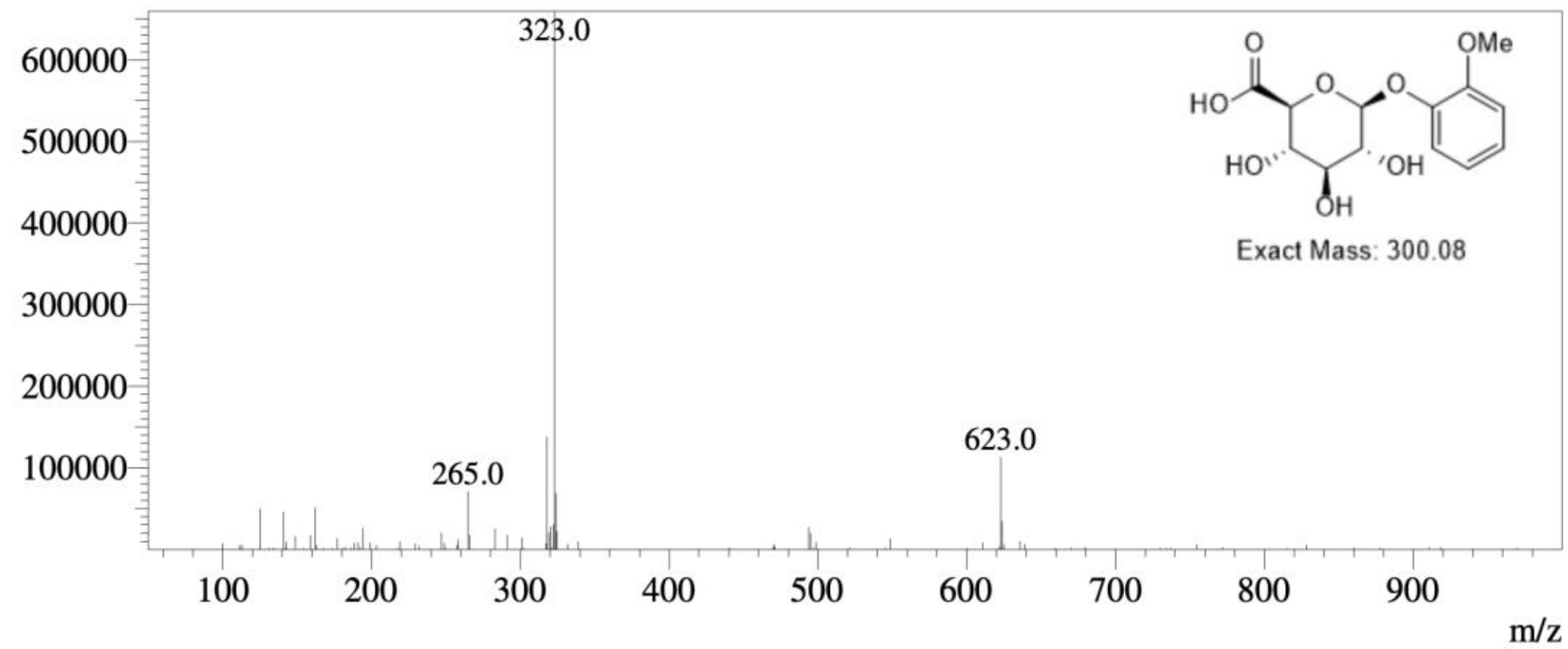

B

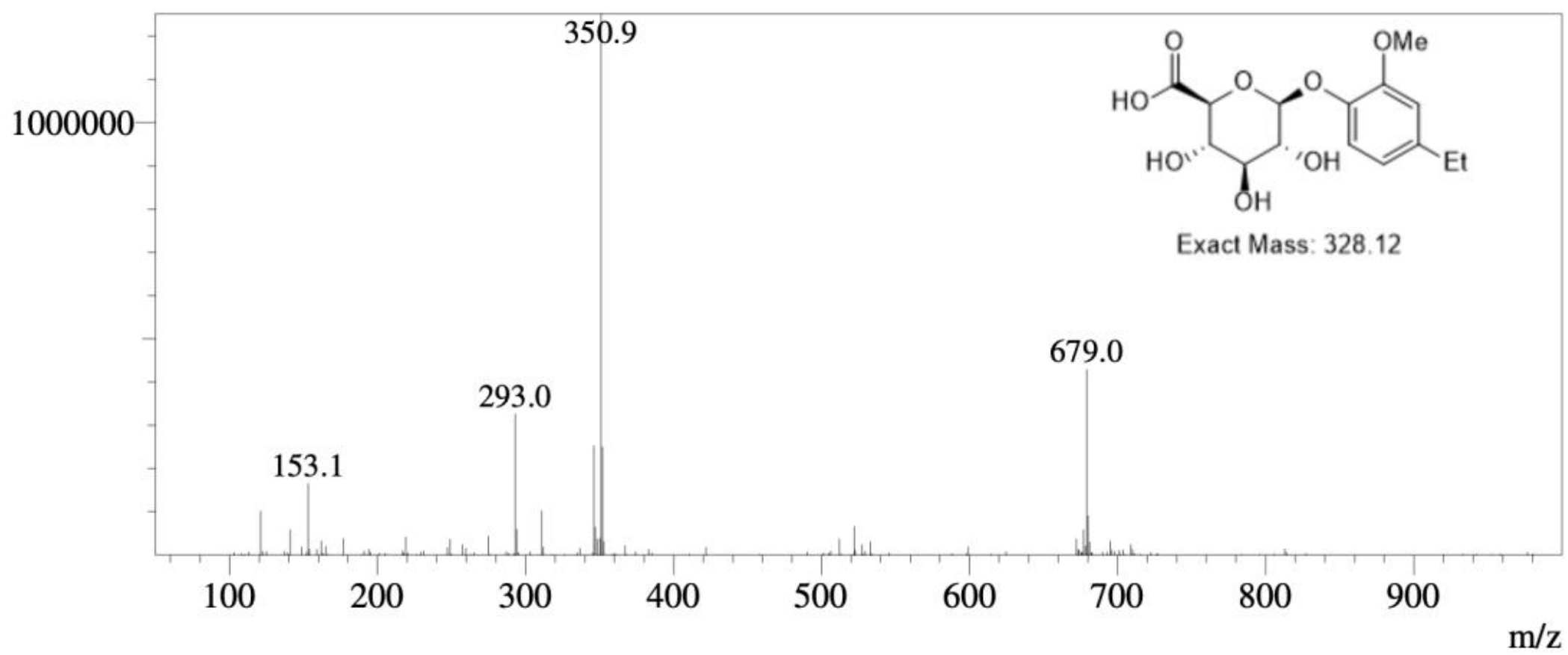

Fig. S2. LC-MS spectra to confirm the mass of (A) GlcA-v124 and (B) GlcA-v152.

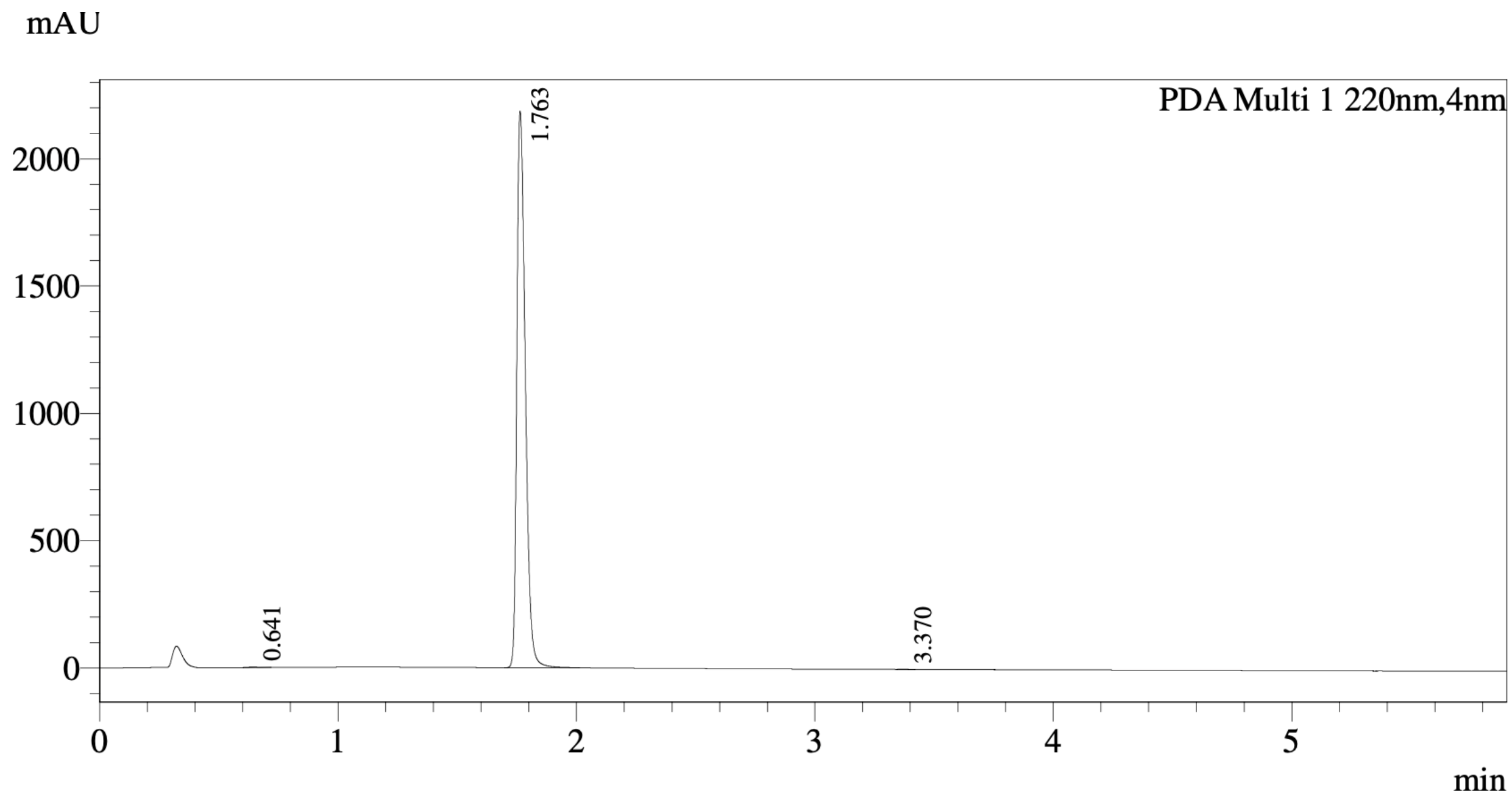

Integration Result

PDA Ch1 220nm

Peak#	Ret. Time	USP Width	Height	Height%	Area	Area%
1	0.641	0.092	1676	0.077	5917	0.110
2	1.763	0.063	2183560	99.884	5389472	99.864
3	3.370	0.044	851	0.039	1424	0.026
Total			2186087	100.000	5396812	100.000

Fig. S3. HPLC chromatogram and peak integration values to characterize the purity of GlcA-v124.

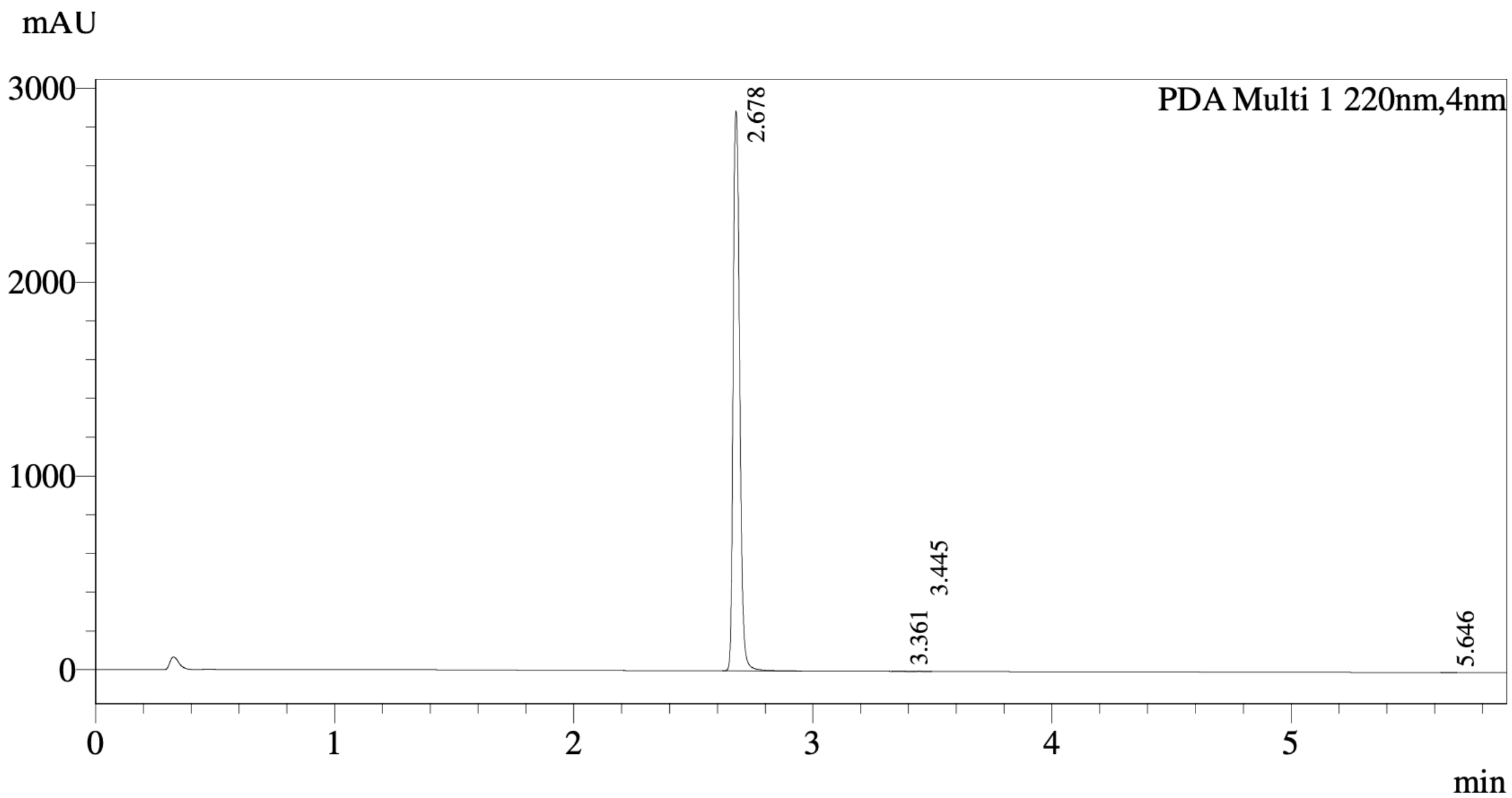

Integration Result

PDA Ch1 220nm

Peak#	Ret. Time	USP Width	Height	Height%	Area	Area%
1	2.678	0.046	2887621	99.888	5494393	99.908
2	3.361	0.042	1843	0.064	3033	0.055
3	3.445	0.040	808	0.028	1242	0.023
4	5.646	0.036	596	0.021	777	0.014
Total			2890867	100.000	5499445	100.000

Fig. S4. HPLC chromatogram and peak integration values to characterize the purity of GlcA-v152.

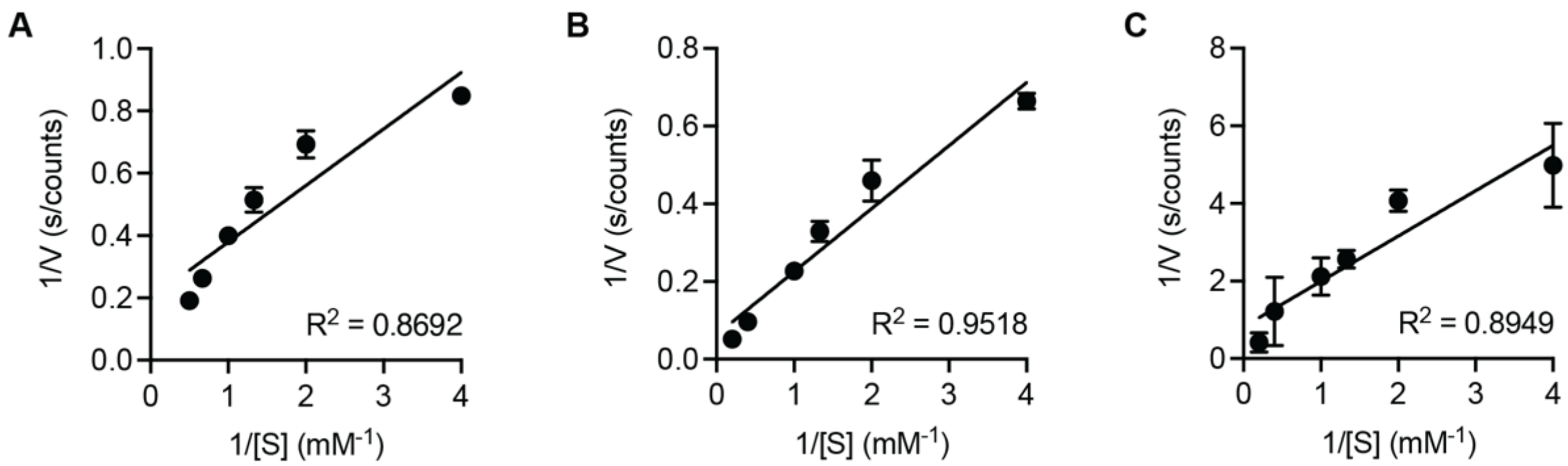

Fig. S5. Lineweaver-Burke plots for GUS reactions with (A) GlcA-v51, (B) GlcA-v124, and (C) GlcA-v152. All data are means ± SD; n = 3 independent experiments.

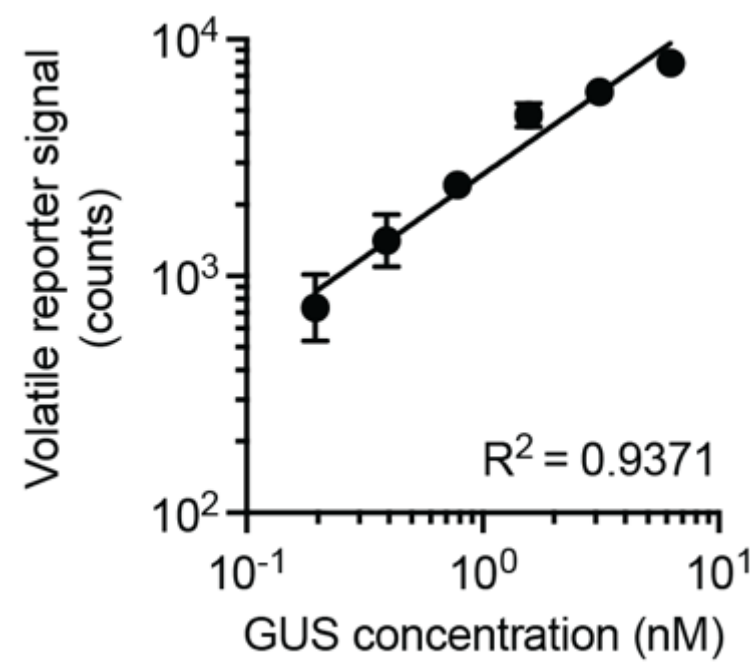

Fig. S6. Volatile reporter signals produced by GlcA-v124 after one-hour reactions with different concentrations of recombinant GUS enzyme. All data are means ± SD; n = 3 replicates; *N* = 2 independent experiments.

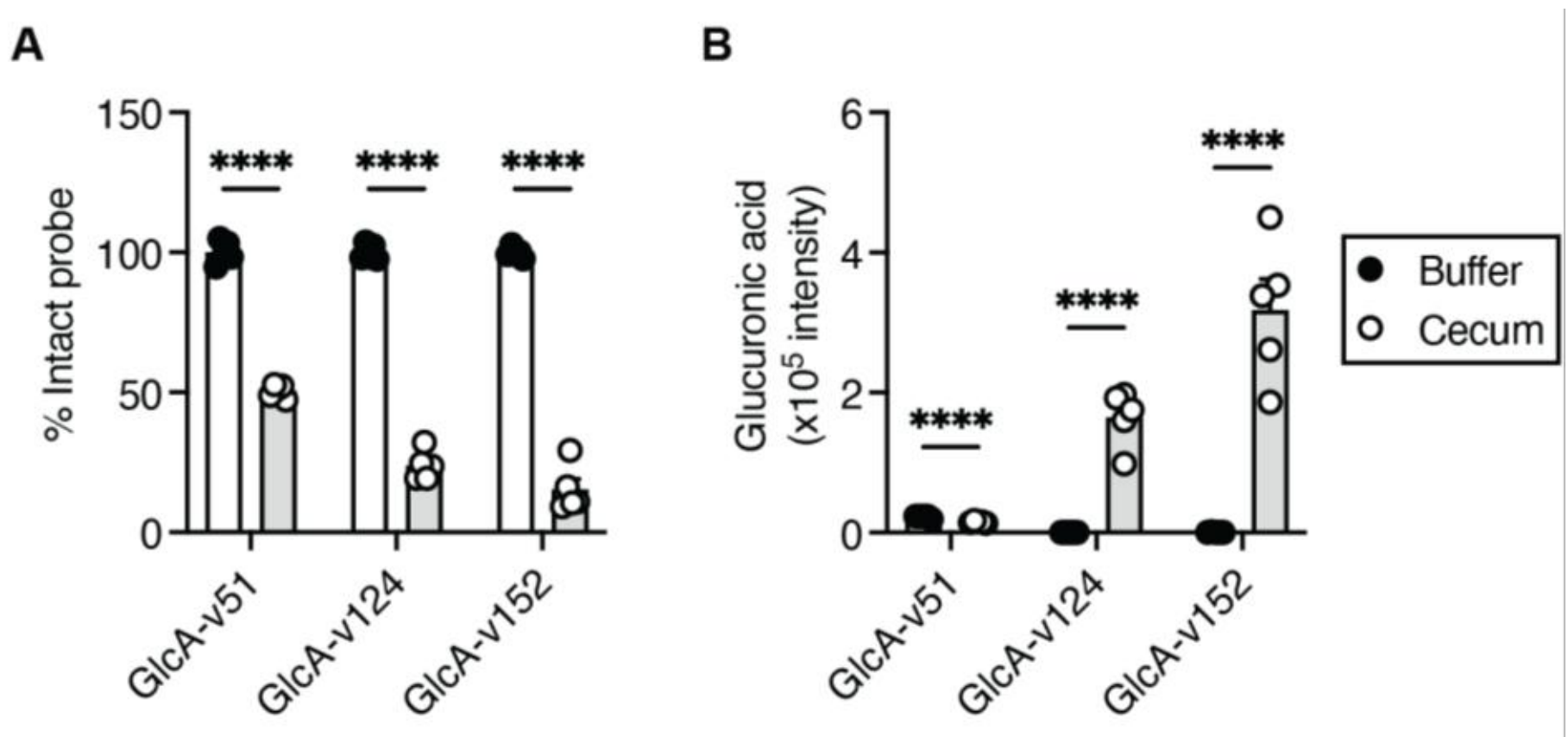

Fig. S7. LC-MS quantification of (A) intact probe and (B) glucuronic acid after one-hour reactions with cecum homogenates. All data are means ± SD; n = 5 mice; unpaired student's t-test, ****p<0.0001.

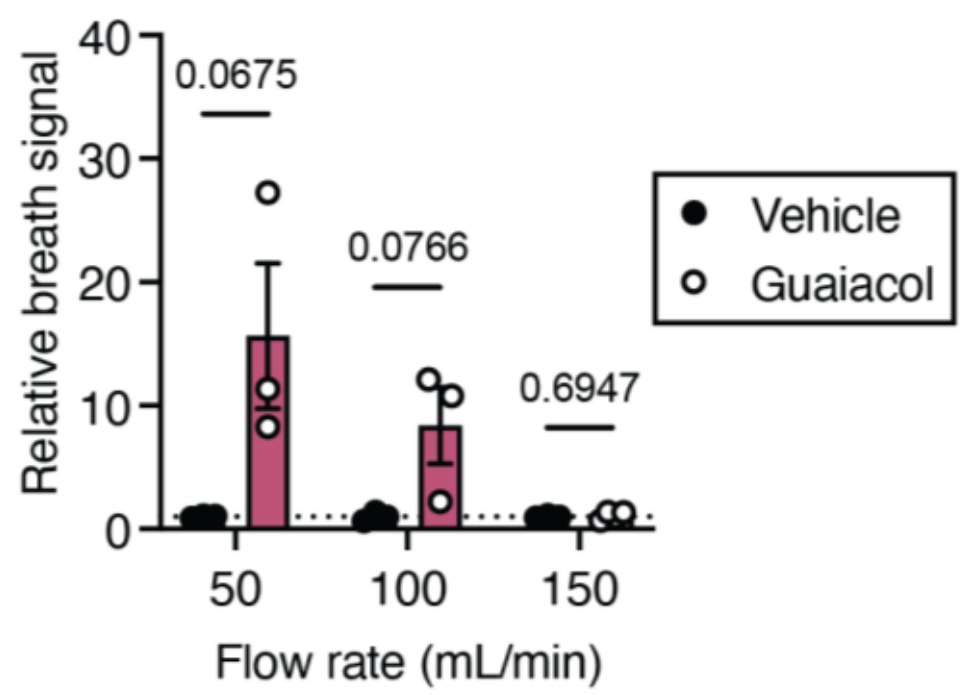

Fig. S8. Guaiacol abundance in breath samples collected at different flow rates. Breath volatiles were collected onto sorbent tubes over a 1h duration immediately following intravenous administration of pure guaiacol. All data are means ± SEM; n = 3 mice; unpaired student's t-test; *p*-values specified in graph.

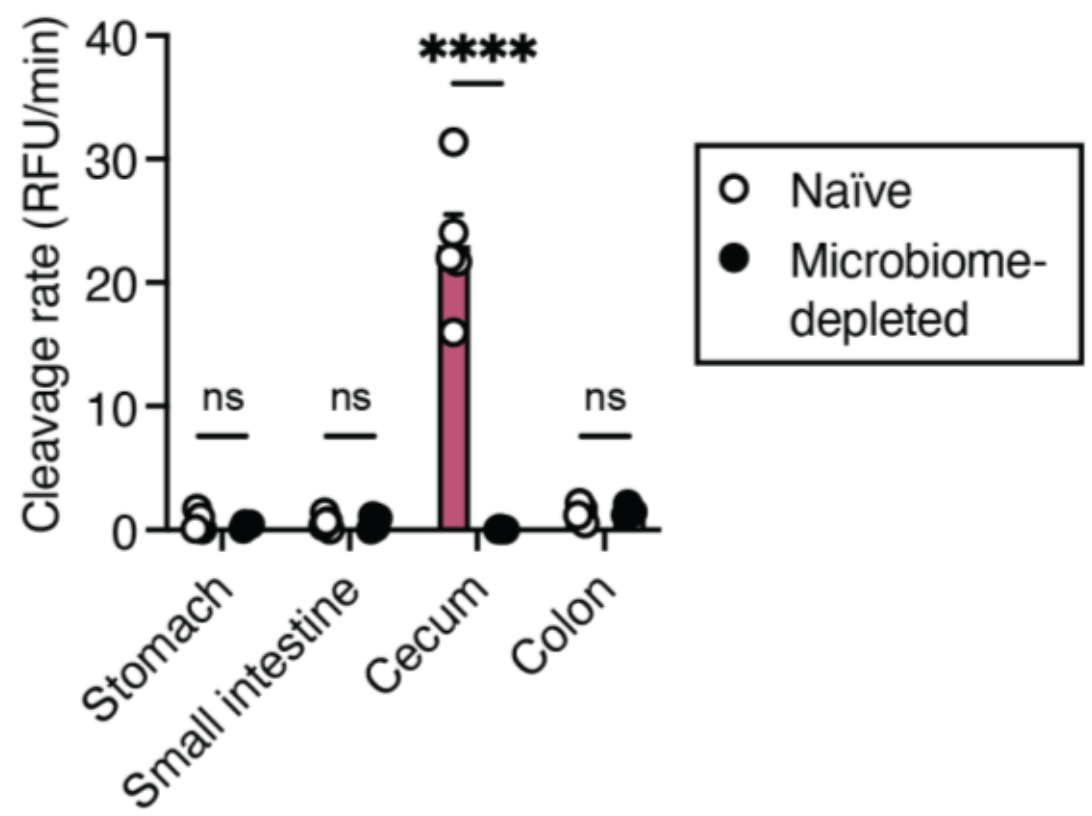

Fig. S9. GUS activity in GI tissue homogenates from naïve mice versus microbiome-depleted mice. GUS activity was measured using FDGlcU. All data are means ± SD; n = 5 mice; unpaired student's t-test, ***p<0.001, ns = not significant; *N* = 2 independent experiments.

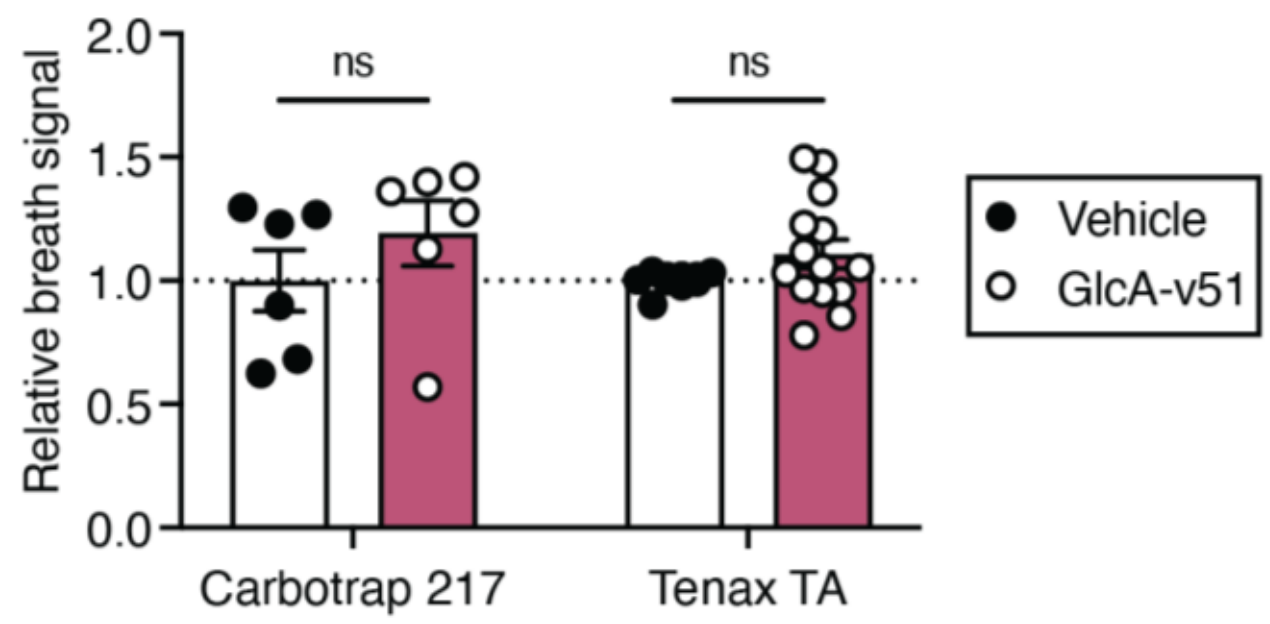

Fig. S10. Comparison of breath signals from GlcA-v51 when breath volatiles are collected using different sorbent tubes. Breath volatiles were collected 2-4 h after oral gavage of GlcA-v51 using Carbotrap® 217 or Tenax® TA sorbent tubes. In contrast to Tenax® TA, which is a single bed sorbent tube, Carbotrap® 217 is a multi-bed sorbent tube for expanded capture of light, polar VOCs like D5-ethanol. No breath signal was observed using either sorbent tube for breath collection after GlcA-v51 delivery. All data are means ± SEM; n = 6-14 mice; unpaired student's t-test, ns = not significant.

Data File S1: Data for main and supplementary figures.
